# Multiplex Site-Directed Gene Editing Using Polyethylene Glycol-Mediated Delivery of CRISPR gRNA:Cas9 Ribonucleoprotein (RNP) Complexes to Carrot Protoplasts

**DOI:** 10.3390/ijms221910740

**Published:** 2021-10-04

**Authors:** Magdalena Klimek-Chodacka, Miron Gieniec, Rafal Baranski

**Affiliations:** Department of Plant Biology and Biotechnology, Faculty of Biotechnology and Horticulture, University of Agriculture in Krakow, AL. 29 Listopada 54, 31-425 Krakow, Poland; miron.gieniec@gmail.com

**Keywords:** Cas9 protein, *Daucus carota*, genome editing, PEG, targeted mutagenesis

## Abstract

The aim of this work was to show an efficient, recombinant DNA-free, multiplex gene-editing method using gRNA:Cas9 ribonucleoprotein (RNP) complexes delivered directly to plant protoplasts. For this purpose, three RNPs were formed in the tube, their activity was confirmed by DNA cleavage in vitro, and then they were delivered to carrot protoplasts incubated with polyethylene glycol (PEG). After 48 h of incubation, single nucleotide deletions and insertions and small deletions at target DNA sites were identified by using fluorescent-PCR capillary electrophoresis and sequencing. When two or three RNPs were delivered simultaneously, long deletions of 33–152 nt between the gRNA target sites were generated. Such mutations occurred with an efficiency of up to 12%, while the overall editing effectiveness was very high, reaching 71%. This highly efficient multiplex gene-editing method, without the need for recombinant DNA technology, can be adapted to other plants for which protoplast culture methods have been established.

## 1. Introduction

The development of a genome editing method, as a consequence of the discovery and research on Clustered Regularly Interspaced Short Palindromic Repeats (CRISPR) and CRISPR-associated (Cas) proteins, was awarded the Nobel Prize in Chemistry in 2020 (https://www.nobelprize.org, accessed on 27 August 2021). The award highlights the significance of understanding the bacterial natural immune system and the potential application of its elements in the biological sciences for genome editing, i.e., for precise site-directed mutagenesis [1]. CRISPR/Cas-based tools, despite their recent development, have been preferentially and widely utilized for genome editing in eukaryotes, including horticultural crops [2], due to their simplicity and efficiency in generating small indel mutations, precise single-base editing, and sequence correction [3].Improved systems utilizing inactivated Cas proteins fused to effectors are also being developed to facilitate regulation of gene expression, targeted epigenetic modification, and in vivo labeling [4,5]. Thus, CRISPR/Cas-mediated genome editing not only revolutionizes basic research but also opens up new possibilities for the development of plants with improved and novel traits of economic and nutritional importance [6], as exemplified by the gene-edited soybean with high nutritional value that has recently been introduced into agriculture, and whose high oleic oil is now available on the US market [7].

In principle, CRISPR/Cas-based tools are used to generate a single- or double-stranded DNA break (DSB) at a precise location. The DNA break results from the cleavage activity of a ribonucleoprotein (RNP) complex consisting of a short oligoribonucleotide, a guide RNA (gRNA), and a type II Cas nuclease, which remains inactive until it forms a functional complex with the gRNA [8]. The RNP complex binds DNA at a site with high sequence homology to the gRNA, next to the conserved protospacer adjacent motif (PAM), and then the Cas protein cleaves the target DNA [9]. The cleavage activity of the RNP complex depends on the type and variant of the Cas protein [10], and the length and structure of the gRNA [11]. The commonly used Cas9 protein cleaves DNA typically 3 nt upstream of the PAM within the gRNA target site and generates a DSB [9]. Site-specific mutations occur as a consequence of cellular mechanisms leading to imperfect DNA repair, mainly through non-homologous end-joining pathways [12].

Successful site-directed mutagenesis requires the delivery of CRISPR/Cas reagents to target cells, which is commonly achieved by designing and multi-step constructing of recombinant DNA vectors expressing Cas proteins and gRNAs, and then transfecting them into the cell [13]. This often results in the integration of foreign DNA within the genome and may affect the expression of native genes [14]. The constitutive expression also favors undesirable off-target mutations, which cumulate over time and lead to mosaicism [15]. Additionally, this approach requires codon optimization for efficient Cas transcription as well as a selection of suitable promoters for the expression of both Cas and gRNA in host cells [16]. Recently, the delivery of preassembled RNP complexes to plant cells without the use of DNA vectors has been reported [17,18]. The delivered RNP complexes induce mutations and are then degraded by cellular enzymes so that they are only transiently active in the host cell, preventing off-target mutagenesis [19]. The resulting mutants are transgene-free, lack foreign nucleic acids, and are undistinguished from spontaneous mutants [20]. Moreover, the chemical synthesis of gRNA makes the whole process independent of recombinant DNA technology, the use of which is bounded by restrictive regulations in most countries [21]. Hence, the delivery of preassembled gRNA:Cas RNP complexes has obvious advantages over the use of DNA vectors expressing Cas and gRNA and is a highly promising approach but requires effective methods of RNP delivery to host cells while ensuring that the complex is active and generates mutations at high rates [22,23]. To date, single RNPs have been delivered either to protoplasts treated with polyethylene glycol (PEG) or to embryos by particle bombardment, and only PEG-mediated protoplast transfection used in selected horticultural species [24]. The efficiency of RNP-induced mutagenesis varied widely and depended on the RNP delivery method, plant species, source of protoplasts, and gRNA sequence [11]. So far, multiplex site-directed mutagenesis by simultaneous delivery of different RNPs has not been reported, except in *Arabidopsis* protoplasts [17].

Here we show a method for efficient multiplex gene editing using three RNP complexes delivered to plant protoplasts in pairs or in triplicate. For this purpose, carrot protoplasts were used as a model, and they were treated with PEG and RNPs containing gRNAs targeting three sites in the same gene. We show the method is highly efficient for multi-targeted site-directed mutagenesis and to generate both small indels at the target sites and whole fragment deletions between any two target sites.

## 2. Results

### 2.1. Validation of the RNP Complex Activity

Three RNP complexes were formed in the tube after incubation of Cas9 with one of the three designed gRNAs:gRNA1:Cas9 (RNP1), gRNA2:Cas9 (RNP2), and gRNA3:Cas9 (RNP3). In vitro DNA cleavage experiments were set up to verify that the gRNAs were correctly designed and targeted to the *F3H* gene and that the already formed RNP complexes exhibited nucleolytic activity. For this purpose, a 367 bp PCR-amplified *F3H* fragment labeled with FAM, was incubated in the presence of each RNP. Cleaved DNA products were detected for all three RNP complexes by fluorescent-PCR capillary electrophoresis (Supplementary Figure S1). They were 324 bp, 205 bp, or 170 bp in length when RNP1, RNP2, or RNP3, were used, respectively, as expected. Only undigested DNA with a length of 367 bp was detected in the control (RNP–). The cleavage efficiency depended on the gRNA present in the RNP complex and was the highest for RNP2, for which half of the DNA molecules were cleaved during 15 min of incubation. The relative cleavage efficiencies for RNP1, RNP2, and RNP3 complexes were 3:9:1, respectively. The presence of the expected cleavage products confirmed that all RNP complexes recognized DNA target sites and were capable of generating DSBs at these sites, and thus they all were suitable for further experiments with living cells.

### 2.2. Single Site Editing

**Figure 1:**
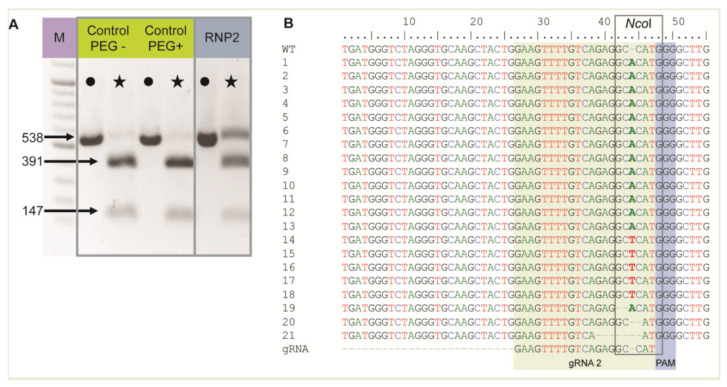
The carrot *F3H* gene fragment edited after delivery of RNP2 to PEG-treated protoplasts. (**A**) DNA of control and RNP2-treated protoplasts. Lanes with the PCR amplified WT 538 bp fragment are marked by dots while lanes with products after incubation with *Nco*I endonuclease recognizing the restriction site at the gRNA2 target site are marked by stars. (**B**) Independent mutant variants of the *F3H* gene aligned to WT and gRNA2 sequences with the *Nco*I restriction sitemarked. M—GeneRuler DNA Ladder Mix (Thermo Fisher Scientific, Waltham, MA, USA).

The RNP2 complex was chosen to show RNP delivery to live protoplasts when incubated with PEG. Two controls without RNPs were also included: (1) untreated protoplasts (PEG–, RNP–) and (2) protoplasts treated with PEG only (PEG+, RNP–). Nested PCR was performed using DNA isolated from protoplasts after PEG and RNP treatment, and the expected 538 bp long *F3H* fragment was detected in all samples, including controls, confirming correct DNA isolation and PCR (Figure 1A). The *F3H* fragment chosen for amplification contained an *Nco*I restriction site in the region targeted by gRNA2. Hence, the amplicons were treated with *Nco*I to determine whether the RNP2 complex generated mutations at this site. For both controls, the restriction site was recognized and the 538 bp long amplicons were completely digested to two fragments of 391 bp and 147 bp (Figure 1A). In the case of RNP-treated protoplasts, these fragments were accompanied by a clearly visible undigested amplicon of 538 bp. These results indicated that mutations occurred at the *Nco*I restriction site in the fraction of protoplasts treated with RNP2, with efficiencies up to 30% estimated from the comparison of band intensities. Undigested fragments were then excised from the gel, sequenced, and aligned in silico with the *F3H* reference. Mutations were mostly identified between the 3rd and 4th nt upstream PAM, i.e., within the *Nco*I restriction site (Figure 1B). The most frequent mutation (86%) was a single nucleotide insertion, either A or T. Small deletions of 1 to 6 nt, and mutations combining deletion and insertion were also identified. The results showed that RNP complexes can be successfully delivered to protoplasts where they induced mutations at the expected location.

### 2.3. Multiplex Editing

**Figure 2:**
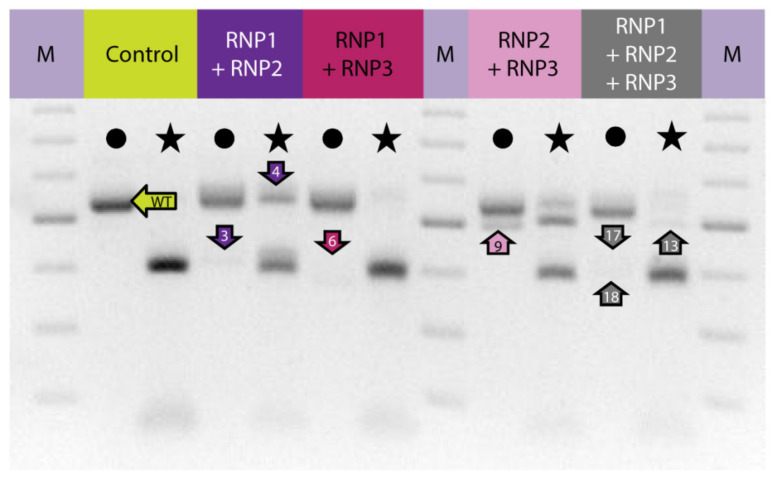
The carrot *F3H* gene fragment before and after delivery of RNP complexes in different combinations to PEG-treated protoplasts. Lanes with the WT 538 bp fragment amplified by PCR are marked by dots while lanes with products after incubation with *Nco*I endonuclease recognizing the restriction site at the gRNA2 target site are marked by stars. Arrows indicate products that were sequenced. M—GeneRuler DNA Ladder Mix (Thermo Fisher Scientific).

To induce concurrent mutations at two target sites, PEG–treated protoplasts were incubated with a mixture of two RNP complexes: RNP1 + RNP2, RNP1 + RNP3, or RNP2 + RNP3. In addition, a mixture of all three RNPs was used to induce mutations simultaneously at three sites. The expected PCR product of 538 bp was obtained for all samples, including the RNP–untreated control while additional shorter products of approximately 390–510 bp were present in samples from RNP-treated protoplasts (Figure 2). When the RNP1 + RNP2 pair was used, the product was approximately 120 bp shorter than the expected 538 bp fragment. This difference corresponded to the 120 nt distance between the canonical Cas9 cleavage sites at the gRNA1 and gRNA2 target sites. Analogous 155 bp and 35 bp shorter products were identified for the other RNP pairs, RNP1 + RNP3 and RNP2 + RNP3, respectively. All three shorter fragments (by approximately 120, 155, and 35 bp) were also identified after incubation of protoplasts with a mixture of all three RNPs. Thus, the presence of shorter than expected products indicates the occurrence of DNA fragment deletions between gRNA target sites.

**Figure 3:**
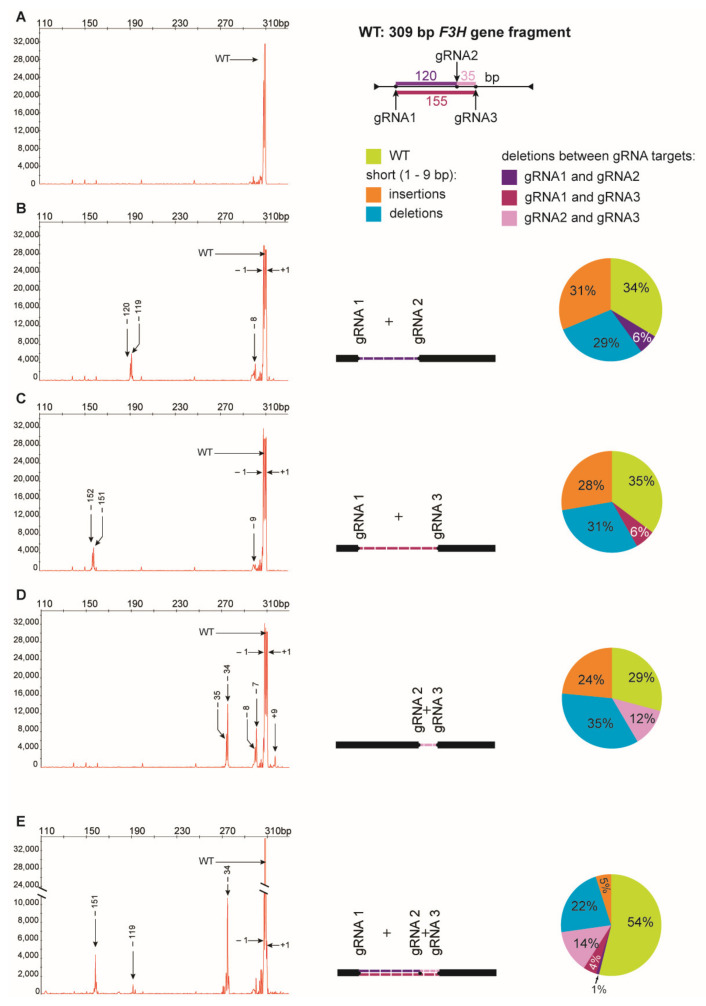
Multiplex editing of the *F3H* gene after delivery of RNPs in pairs or in triplicate. The size of amplified *F3H* gene fragments was determined by fluorescent-PCR capillary electrophoresis. The following combinations of RNPs were used: (**A**) no RNP, (**B**) RNP1 + RNP2, (**C**)RNP1 + RNP3, (**D**) RNP2 + RNP3, (**E**) RNP1 + RNP2 + RNP3. Numbers indicate mutation size: (+) insertions, (−) deletions. Drawings represent deletions of fragments between two gRNA targets. The pie charts show the mutation frequency calculated from the peak area.

**Figure 4:**
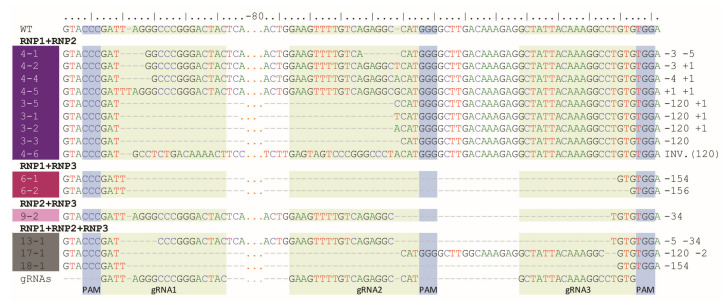
Example mutant variants of the *F3H* gene aligned to WT and gRNA sequences and obtained after RNPs delivery in pairs or in triplicate.

Digestion of PCR products with *Nco*I resulted in 391 bp and 147 bp fragments in control and RNP-treated samples while undigested products were identified in samples obtained from protoplasts treated with RNP mixtures containing RNP2 (Figure 2). Visual comparison of band intensities in the gel indicated that a large fraction of the PCR amplicons remained uncut due to mutations induced at the *Nco*I restriction site, particularly for RNP1 + RNP2 and RNP2 + RNP3 pairs. Accurate determination of fragment sizes was done using the fluorescent-PCR capillary electrophoresis after amplification of a 309 bp *F3H* fragment with ROX-labeled primers. For the control, a single peak of fluorescent signal was detected at 309 bp confirming the presence of the whole, unmodified DNA amplicon (Figure 3A). Samples from protoplasts treated with the RNP1 + RNP2 pair gave complex fluorescent signals (Figure 3B). The position of the peaks indicated that the reaction mixture contained DNA molecules of different lengths, i.e., the unmodified 309 bp fragment was accompanied by fragments with single nucleotide insertions and one to several nucleotide deletions. Additionally present were distinct peaks of much shorter products that indicated deletions of 119 bp and 120 bp, presumably due to the elimination of the whole fragment between gRNA1 and gRNA2 targets. Sanger sequencing confirmed that the short insertions and deletions occurred exactly at the expected gRNA1 and gRNA2 target sites, 3 nt next to the PAMs, and in close proximity to them (Figure 4). Deletions of 120 bp between cleavage sites in both gRNA1 and gRNA2 target sites were also confirmed. Sequencing also revealed a mutant with a perfect inversion of the whole 120 bp fragment between the target sites (Figure 4).

Analogous results were obtained for RNP1 + RNP3 and RNP2 + RNP3 pairs except that *Nco*I restriction occurred only when the RNP2 complex was used (Figure 2). The fluorescent-PCR capillary electrophoresis showed the presence of single nucleotide insertions and deletions, other short (up to 9 nt) insertions and deletions, and long deletions (151–152 bp and 34–35 bp); the latter presumably indicating the elimination of the whole fragments between gRNA targets (Figure 3C,D), which was further confirmed by sequencing (Figure 4).

Multiplex editing was also verified after treatment of protoplasts with a mixture of all three RNPs. As above, some PCR products were shorter than WT and *Nco*I treatment resulted in uncut products (Figure 2). Distinct fluorescence peaks were also detected, the location of which indicated single-nucleotide and short indel mutations as well as the elimination of 151 bp, 119 bp, and 33–35 bp fragments (Figure 3E). Sequencing confirmed deletions of the whole fragments between cleavage sites targeted by any combination of two gRNAs: gRNA1 and gRNA2, gRNA1 and gRNA3, gRNA2 and gRNA3 (Figure 4).

The peak areas are proportional to the number of fluorescing DNA fragments thus they were compared to estimate the proportion of mutants and the proportion between different types of mutations. Mutations occurred at a high frequency ranging from 65% to 71%, independent of which RNP pair was delivered to the protoplasts (Figure 3). Among the mutation types, single nucleotide deletions (32–39%) and insertions (31–47%) were the most frequent. Deletions of the whole fragment between gRNA1 and gRNA2 targets (119–120 bp), and between gRNA1 and gRNA3 targets (151–152 bp) occurred with similar frequency, 9% and 10%, respectively. Deletions of shorter fragments (33–35 bp) between gRNA2 and gRNA3 targets occurred more frequently (17%). When all three RNPs were used simultaneously, mutations occurred with a frequency of 46%. Single nucleotide insertions were less frequent (11%), compared to treatments with any pair of RNPs, while the frequency of whole fragment deletions between gRNA2 and gRNA3 targets (33–35 bp) was twice as high (30%). In contrast, the frequency of whole fragment deletions between gRNA1 and gRNA2 targets (120 bp) was low (2%).

## 3. Discussion

We have demonstrated a complete protocol for efficient multiplex gene editing by direct delivery of gRNA:Cas9 RNP complexes to protoplasts using carrot as a model species for extensive biotechnology-related research [25]. The three RNP complexes were designed to target three sites (35, 120, and 155 bp apart) in the *F3H* gene, whose functional knockout was previously demonstrated by inducing mutations in carrot using CRISPR/Cas9 DNA vectors [2,26] and in petunia using single RNPs [27]. In this work, multiple RNP complexes were formed by simply incubating a commercial Cas9 protein with a laboratory synthesized gRNA, thus facilitating the entire process in-house. Proper gRNA design and RNP formation, and RNP complexes activity were verified prior to protoplast transfection by performing in vitro DNA cleavage. Fluorescent-PCR capillary electrophoresis was applied for a fast and accurate determination of the size of the cleaved products [28], and the detected fluorescent signals univocally confirmed the correct preparation of the RNP complex. In earlier reports, different ratios of Cas9 and gRNA were used to form RNPs, which ranged from 1:1 to 1:7, without clear recommendations [27,29,30]. However, the efficiency of mutagenesis was more dependent on the choice of target locus than on the ratio of RNP components [31]. In our protocol, a molar ratio of Cas9 and gRNA of 1:5 allowed successful RNP formation. RNPs differed in cleavage activity, which corroborates opinions that the seed sequence of the gRNA affects editing efficiency [32]. However, subsequent experiments with protoplasts showed that the use of RNPs highly differing in cleavage activity resulted in similar frequencies of mutants. Hence, low cleavage activity determined in vitro should not be considered as limiting effective mutagenesis.

For most crop plants the reported mutation efficiencies after RNP delivery were well below 30% and higher efficiencies than 50% were obtained only when the estimation was done using potato [33] and *Arabidopsis* plants regenerated from RNP-transfected protoplasts [17]. However, it should be emphasized that different methods can be used to estimate mutation efficiency making direct comparisons ambiguous. Nevertheless, the mutation efficiency obtained in this work reached 71%, which is one of the highest reported to date [34]. Such high efficiency was partly due to the simultaneous use of RNPs cleaving two independent targets and was more than double that of using a single RNP. In contrast, the use of three RNPs did increase the number of mutants. This could be explained by the simultaneous generation of DSBs at both flanking target sites, gRNA1 and gRNA3, leading to the deletion of the whole fragment spanning between these target sites and containing the third target site (gRNA2), which was either no longer available or mutated before the fragment was eliminated. Deletions of long, 120, and 151 bp, DNA fragments were detected in about 10% of mutants, and deletions of shorter fragments (34 bp) were almost two times more frequent. The delivery of three RNPs to the protoplasts also revealed that the shortest fragment between the two targets was preferentially deleted. These observations indicate that the 34 bp distance between the two gRNA targets does not hinder RNP binding and DNA cleavage, even when both gRNAs are complementary to the same DNA strand. Single nucleotide deletions and insertions causing a shift in the reading frame were the most frequent mutations accounting together for 49% to 82% of mutants. Inversion of a 120 bp fragment between two gRNA targets exemplifies complex rearrangements that may occur when several sites are targeted simultaneously, and which are rarely reported [35]. RNP pairing has several beneficial consequences. It ensures successful mutagenesis even if one RNP fails, increases the probability of mutation when both RNPs are active, and enables deletion of a long stretch of DNA, which in turn increases the chance of gene knockout if desired. The use of three, or more, RNPs may be highly appreciated when used for site-directed mutagenesis at locations scattered in the genome, however, it may be limited when the targeted sites are in close proximity, and when eventual long fragment deletions may result in the elimination of some target sites.

The high editing efficiency reported here was obtained within the first 48 h after RNP delivery, i.e., well before the first cell divisions. Carrot protoplasts start to recover their cell wall no earlier than three days after the initiation of culture, and the first cell divisions are observed within the following days [36]. Pre-assembled RNP complexes are delivered to cells and are not synthesized de novo, so their number in the cell decreases over time due to their natural degradation and separation to daughter cells at each subsequent cell division [34,37]. Moreover, editing efficiency reaches a plateau in prolonged culture [38]. In consequence, further RNP-induced mutations during later stages of culture are not expected and the probability of mosaicism is reduced [17].

## 4. Material and Methods

### 4.1. Plant Material

Carrot (*Daucus carota* L. subsp. *sativus* Hoffm.) callus line derived from ‘Koral’ variety. Callus was maintained in Petri dishes with 0.2% phytagel (Sigma, St. Louise, MO, USA) solidified BI mineral medium composed of Gamborg B5 salts with vitamins (Duchefa, Haarlem, The Netherlands) supplemented with 1 mg/L 2,4-dichlorophenoxyacetic acid (2,4-D), 0.025 mg/L kinetin (Duchefa), and 30 g/L sucrose; pH 5.8 and incubated at 26 °C in the dark.

### 4.2. gRNA Design and Synthesis

Three gRNAs (Table 1) targeting the second exon of the carrot *F3H* gene (NCBI Acc. No. AF184270.1) were designed using CasOT software [39], which also allows the identification of potential off-target regions in the carrot genome. The specificity of the gRNA was additionally verified using Cas-OFFinder software with a 1 nt mismatch allowance [40]. The *Nco*I restriction site was present in the gRNA2 target sequence in close proximity to PAM. Synthesis of gRNA was performed using the GeneArt ™ Precision gRNA Synthesis Kit (Invitrogen, Carlsbad, CA, USA) following the manufacturer’s recommendations. Briefly, 0.3 µM of target oligonucleotides were PCR-assembled into the gRNA DNA template. For this purpose, 12.5 µL of Phusion HF PCR Master Mix was mixed with 1µL Tracr Fragment with T7 Primer and water to a volume of 25 µL. A two-step PCR assembly was performed with the following parameters: initial denaturation of 10 s at 98 °C, 32 cycles of denaturation (5 s; 98 °C), and annealing (15 s; 55 °C) followed by the final extension at 72 °C for 1 min. In vitro transcription was performed at 37 °C for 4 h using TranscriptAid Enzyme Mix. Finally, the synthesized gRNAs were purified on the attached columns and sequenced, without cloning, using a primer complementary to the tracrRNA sequence of gRNA (Table 2; gRNA_seq).

### 4.3. PCR Fragment Digestion In Vitro

The PCR reaction was set up as previously described [26], with modifications: annealing at 60 °C and reaction volume of 20 µL. The reaction was set up with the F3H_F and F3H_R_FAM primers (Table 2), of which the reverse primer was labeled with the 6-carboxyfluorescein (FAM) fluorescent dye. The PCR products were separated in a 1% agarose gel to confirm the correct length of the amplicons and then purified using the Wizard^®^ SV Gel and PCR Clean-Up System kit (Promega, Madison, WI, USA). The concentrations of PCR products and gRNA were measured using the NanoDrop spectrophotometer (Thermo Fisher Scientific, Waltham, MA, USA). In vitro digestion of the PCR fragment was carried out in a Cas9:gRNA:PCR reaction mixture with a molar ratio of 10:10:1, respectively. For this purpose, Cas9 and one of the three gRNAs were mixed in the presence of buffer 3.1 (NEB), and the PCR product was added after 10 min incubation at 25 °C. Digestion was carried out at 37 °C for 15 min, after which 1 µL of Proteinase K (Sigma Aldrich) was added. PCR fragments were separated using capillary electrophoresis (3730XL DNA Analyzer, Applied Biosystems, Foster City, CA, USA) combined with the detection of fluorescent dyes. DNA fragment lengths were determined using the GeneScan™ 500 LIZ size marker (Applied Biosystems) and visualized using PeakScanner v.2.0 software (Thermo Fisher).

### 4.4. Protoplast Isolation and PEG-Mediated Transformation

Cas9 proteins (EnGenTM Cas9 NLS, New England BioLabs, Ipswich, MA, USA) were premixed with the synthesized gRNA at a molar ratio of 1:5 in 1x Cas9 Nuclease Reaction Buffer (20 mM HEPES, 100 mM NaCl, 5 mM MgCl_2_, 0.1 mM EDTA; pH 6.5) and sterile, distilled water was added to the final volume of 20 µL. The mixture was incubated at 37 °C for 15 min to allow the formation of the RNP complex. The whole volume of assembled RNP complex was carefully added to 200 µL of isolated and counted protoplasts (approximately 16 × 10^5^ protoplasts) and gently mixed by tapping. Freshly prepared and sterilized 220 µL of polyethylene glycol (PEG) solution (40% PEG 4000, 0.4 M mannitol, 1 M CaCl_2_, milli-Q water) was added and gently mixed by pipetting. When two or three gRNAs were used simultaneously, the RNP complexes were prepared separately and then added to the protoplast suspension. The mixture was incubated for 20 min at room temperature (RT) and then washed three times in W5 solution by centrifugation (100× *g* for 3 min). The final protoplast pellet was resuspended in 300 µL of W5 solution and incubated at 26 ˚C for 48 h in the dark.

### 4.5. PCR and Restriction Fragment Analysis

Genomic DNA was extracted from protoplasts 48 h after PEG treatment using the CTAB method with modifications [41]. Protoplasts were centrifuged (3 min, 3300× *g*) in 2 mL eppendorf tubes. After decanting the supernatant, the pellet was ground in the MM400 mixer mill (Retsch GmbH, Haan, Germany) using 3 mm balls in the presence of 100 µL of CTAB buffer for 3 min at RT. To detect mutations in the *F3H* gene, a nested PCR was performed using the primer pairs listed in Table 2, followed by digestion with the *Nco*I endonuclease as described previously [26]. Amplified fragments were separated in a 2% agarose gel. Undigested fragments were eluted from the gel and purified using the Promega Wizard^®^ SV gel and PCR clean-up system (Promega). After cloning into pGEM plasmids (Promega) and subsequent genetic transformation into *E. coli*, plasmids were isolated using the GeneJET Plasmid Miniprep Kit (Thermo Fisher) according to the manufacturer’s instructions. Samples were subjected to Sanger sequencing using standard Sp6 and T7 primers. Reads were manually aligned to the reference sequence of the *F3H* gene using BioEdit v. 7.2.5 software [42].

To detect indel mutations, PCR was performed as previously described [26] using the 5’-end ROX labeled F3H_GS_Rox and F3H_RM primer pair (Table 2) with the annealing temperature set to 60 °C. Products were diluted 10-fold and separated using capillary gel electrophoresis as described above.

## 5. Conclusions

The paper presents a novel method for delivering RNPs to carrot protoplasts and highly efficient gene editing at multiple target DNA sites that may be applied to generate various mutations, including deletions of fragments of predictable length, when more than one RNP is delivered simultaneously to protoplasts. This method can be adapted to other species for which protoplast culture methods have been established.

## Figures and Tables

**Table 1:** Selected gRNAs sequences with PAM type located upstream used for carrot *F3H* gene editing.

Name	Sequence 5′–3′	Length	PAM
gRNA1	GTAGTCCCGGGCCCTAATC	19	GGG
gRNA2	GAAGTTTTGTCAGAGGCCAT	20	GGG
gRNA3	GCTATTACAAAGGCCTGTG	19	TGG

**Table 2:** Primers used for PCR and Sanger sequencing.

Name	Sequence 5′–3′	Annealing Temp. (°C)	Expected Product Length (bp)	Purpose
F3H_F	GCAAGATTGGCGAGAGATAG	60	367	In vitro digestion
F3H_R_FAM	AGTGATCCAGGTTTTTCCGC			
F3H_FO	GAGAAACTCCGGTTCGATATG	56	709	I step nested PCR
F3H_RO	CTGAACAGTGATCCAGGTTT			
F3H_FM	CGTGTTATCGTTGGGATCGG	56	538	II step nested PCR
F3H_RM	AGCAAGAGCGTAATTGTGCC			
F3H_F_ROX	GCAAGATTGGCGAGAGATAG	60	309	fluorescent PCR
F3H_RM	AGCAAGAGCGTAATTGTGCC			
gRNA_seq	GACTCGGTGCCACTTTTTCA	-	-	gRNA sequencing after in vitro transcription

## Data Availability

Not applicable.

## Supplementary Materials

The following are available online at https://www.mdpi.com/article/10.3390/ijms221910740/s1.
