# Supplementary material for: Multiplex Site-Directed Gene Editing Using Polyethylene Glycol-Mediated Delivery of CRISPR gRNA:Cas9 Ribonucleoprotein (RNP) Complexes to Carrot Protoplasts
Source: Int J Mol Sci. 2021 Oct 4;22(19):10740. doi: 10.3390/ijms221910740 (PMC8509836; doi:10.3390/ijms221910740)
Supplement: Supplementary file 1 [file ijms-22-10740-s001.zip › ijms-1394665-supplementary.pdf]

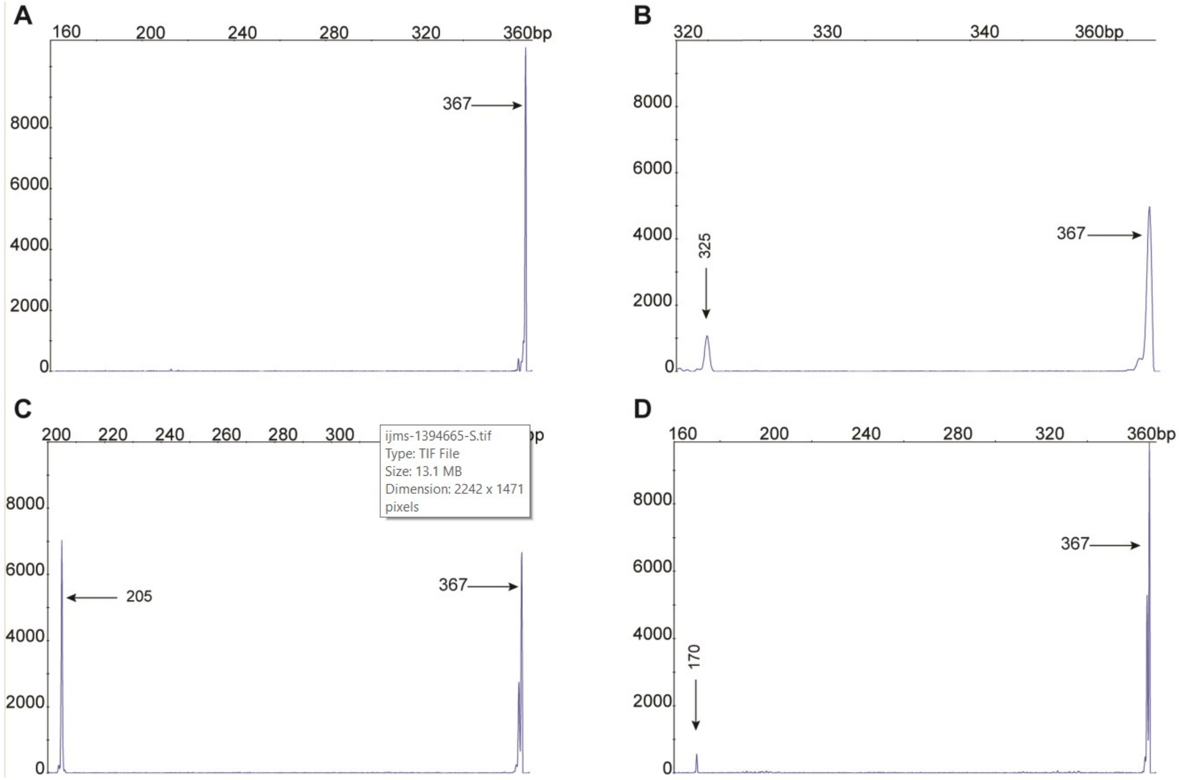

Figure S1. Cleaved fragments after incubation of a 309 bp long *F3H* gene fragment with one of the pre-assembled RNP complexes and detected by using a fluorescent-PCR capillary electrophoresis. RNPs used for in vitro DNA cleavage: (**A**) no RNP, (**B**) RNP1, (**C**) RNP2, (**D**) RNP3.
